# Change in medical practice over time? A register based study of regional trends in hysterectomy in Finland in 2001–2018

**DOI:** 10.1186/s12905-021-01386-2

**Published:** 2021-06-14

**Authors:** Tiina Vikstedt, Martti Arffman, Satu Heliövaara-Peippo, Kristiina Manderbacka, Eeva Reissell, Ilmo Keskimäki

**Affiliations:** 1 University of Helsinki, P.O. Box 4, 00014 Helsinki, Finland; 2 Welfare State Research and Reform Unit, Finnish Institute for Health and Welfare (THL), P.O. Box 30, 00271 Helsinki, Finland; 3 Department of Obstetrics and Gynaecology, Helsinki University Central Hospital, P.O. Box 100, 00029 HUS, Finland; 4 Faculty of Social Sciences, Tampere University, 33014 Tampere, Finland

**Keywords:** Medical practice variation, Hysterectomy, Health services research, Register-based research

## Abstract

**Background:**

A persistent research finding in Finland and elsewhere has been variation in medical practices both between and within countries. Variation seems to exist especially if medical decision making involves discretion and the best treatment cannot be identified unambiguously. This is true for hysterectomy when performed for benign causes. The aim of the current study was to investigate regional trends in hysterectomy in Finland and the potential convergence of rates over time.

**Methods:**

We used hospital discharge register data on hysterectomies performed, diagnoses, age, and region of residence to examine hospital discharges for women undergoing hysterectomy in 2001–2018 among total female population aged 25 years or older in Finland. We examined hysterectomy rates among biannual cohorts by indication, calculated age-standardised rates and used multilevel models to analyse potential convergence over time.

**Results:**

Altogether 131,695 hysterectomies were performed in Finland 2001–2018. We found a decreasing trend, with the age-adjusted overall hysterectomy rate decreasing from 553/100,000 person years in 2001–2002 to 289/100,000 py in 2017–2018. Large but converging regional differences were found. The correlations between hospital district intercepts and slopes in time ranged from − 0.71 to − 0.97 (*p* < 0.001) suggesting diminishing variation.

**Conclusions:**

Our findings demonstrate that change in hysterectomy practices and more uniformity across regions are achievable goals. Regional variation still exists suggesting differences in medical practices.

## Background

A persistent research finding in Finland and elsewhere has been variation in medical practices both between and within countries. In a relatively recent systematic review, Corallo et al. [1] found a large number of studies with regional variation within OECD countries covering hospital admissions due to several chronic conditions and elective surgical procedures. Some of the variation seems to be unwarranted, as these differences do not vanish when regional differences in need are taken into account, thus potentially reflecting inequity or inefficiency in the system [2]. Variation seems to exist especially if medical decision making involves discretion and the best treatment cannot be identified unambiguously [3]. This is true for hysterectomy when performed for benign causes. While the rate of hysterectomy has decreased especially in OECD countries, it is still one of the most common gynaecological procedures [4–14]. In Finland hysterectomy rates were high compared to many other countries in the 1990s, but a substantial decrease has been reported since the beginning of the 2000s [15] and the rates are now comparable to other Nordic countries.

While most studies investigating differences in use of hysterectomy have been longitudinal, studies on temporal variations within one country are rare. Additionally, many of the studies examining regional variation do so with total hysterectomy rates [5, 7, 11, 12] and only a few separate between rates for benign and malign conditions [8, 13].

In Finland, earlier studies concerning regional variation in hysterectomy are rare. An earlier study reported large regional variation in overall hysterectomy rates in the late 1980s, and decrease in it [16]. It has not been examined how variation has evolved since. In terms of medical practice variation it is important to examine regional variation in hysterectomy rates for malign and benign conditions separately, as there are non-invasive care options in many of the benign indications and thus more discretion on whether to operate or not.

The Finnish health-care system provides a good case for examining variations in hysterectomy as the system is universal in coverage and therefore, in general, supports equity in access to health-care according to need [17]. The system is mainly financed by tax revenues and user-fees are generally low. The system supports evidence based care as there are accepted guidelines for the treatment of altogether 106 conditions including guidelines for treatment of several benign causes related to hysterectomy in the National Current Care guidelines system [18].

The aim of this study was to investigate change in use of hysterectomy in Finland from 2001 to 2018. We concentrate mainly on hysterectomies performed for benign indications. We further evaluate whether the potential decrease in differences suggested by earlier studies has occurred in Finland as well, and whether it occurred in all indications or just some of them.

## Methods

We used Finnish Care Register for Health Care (HILMO) data from 1 January 2001 to 31 December 2018 to assess changes in the use of hysterectomy. The register covers all hospital discharges in all public and private hospitals in Finland. The study population consisted of all female residents in Finland aged 25 or older between 2001 and 2018. Population at risk was defined as female mean population in each age group and hospital district. Hospital districts were based on municipalities according to residence. Persons whose permanent residence was outside Finland and those in long-term care were excluded from the analyses.

We defined hysterectomies using Nomesco operational codes (LCC00, LCC01, LCC10, LCC11, LCC20, LCC96, LCC97, LCD00, LCD01, LCD04, LCD10, LCD11, LCD30, LCD31, LCD40, LCD96, LCD97, LEF13, LEF14). We categorized indications for hysterectomy according to diagnostic codes from the Finnish version of ICD-10 and classified hysterectomies into seven categories: leiomyoma of uterus (D25), gynecological malignancy, malignant neoplasm and in situ neoplasms of female genital organs (C51-C58, D06, D07, D39), abnormal uterine bleeding (AUB; N91-N93, N95), endometriosis (N80), genital prolapse (N81), and “other”, including the remaining gynaecological diagnoses. Any record of primary malignant neoplasm and in situ neoplasm of female genital organs was assigned as a primary diagnosis independent of its position in the list of diagnoses. If no diagnosis of cancer or in situ neoplasm was recorded, the diagnosis listed first was designated as the indication for hysterectomy. Peripartum hysterectomies (MCA30, MCA33, MCW00) were not included in the study. However, they are rare in Finland and should therefore, not have a significant effect on the results of the current study.

To analyse regional and temporal variations, age-adjusted rates per 100,000 person years (py) were estimated using a direct method of standardisation with the standard population being female residents aged 25 or over in Finland in 2018. Age-standardised hysterectomy rates were calculated separately for each indication. Age was classified into four age groups: 25–49 years, 50–59 years, 60–69 years and 70 or over.

Regional and temporal variations in hysterectomy rates were analysed in 20 hospital districts. They are owned by federations of municipalities and responsible for organisation of public specialised care for the residents of their area.

To estimate regional heterogeneity in practices we calculated systemic component of variation (SCV) between health care regions as a descriptive measure of annual variation in procedure rates. It is a relative measure that indicates whether the variation found is larger than could be expected by chance.

To examine consistency in regional variation in use of hysterectomy from 2001 to 2018, the autocorrelations per region and per indication were computed according to the method described by Westert et al. [2]. The annual hysterectomy rates by different indications within each region during the 18-year study period cannot be assumed to be independent measurements as rates can be systematically higher or lower in some regions e.g. due to physician preferences. The small autonomous region of the Åland Islands was excluded from the analyses.

The trend analysis was performed for biennial age-standardised hysterectomy rates with fixed effects for intercept and a polynomial function of time and random effects for intercept and slope varying across regions. Next, z scores were calculated for annual hysterectomy rates. This was done to capture the general national trend in hysterectomy rate over years and allow for comparison of regional rates over different indications in comparable scale. We used biennial rates to acquire more consistency into analysis. In the first analytical step all diagnostic or procedural categories were simultaneously used in operational models similar to above-mentioned model. In these models, the time trend of spatial variation is given by the correlation of the intercept and the slope in time at level 2. We interpreted a statistically significant negative correlation as decrease of regional variation with higher than average random intercepts linked with larger than average decrease in random slopes and vice versa. In the second analytical step a similar two-level model was run for each hysterectomy indication separately. Statistical analyses were performed using SAS 9.4 (SAS Institute Inc, Cary, NC, US).

## Results

A total of 131,695 hysterectomies performed in Finland 2001–2018 were included in the analyses. Descriptive statistics of hysterectomies in 2001–2002 and 2017–2018 are summarized in Table 1. In 2001–2002 the total number of hysterectomies performed was 21,305 and 11,812 in 2017–2018, which corresponds to a decline in age-standardised hysterectomy rates from 553/100,000 py in 2001–2002 to 289 in 2017–2018 (− 49%).

**Table 1:** Hysterectomy rate per 100,000 person years by age and indication in Finland in 2001–2002 and 2017–2018

	Rate in 2001–2002	(N)	Rate in 2017–2018	(N)	Change (%) in rate
*Age*					
25–49	561	9859	256	4257	− 54
50–59	837	6260	389	2841	− 54
60–70	523	2735	294	2223	− 44
70+	341	2451	265	2490	− 22
Crude rate	569	21,305	289	11,812	− 49
Age-adjusted rate*	553		289		− 48
*Indication*					
Leiomyoma*	212	8692	61	2513	− 71
Malignancy*	60	2045	52	2108	− 14
Genital prolapse*	111	3895	81	3325	− 27
Endometriosis*	25	1067	14	572	− 45
AUB*	56	2347	39	1592	− 31
Other diagnoses*	87	3259	42	1701	− 51

*Age-adjusted by using the age structure in 2018 data as the standard

Over the study period the hysterectomy rates decreased in all age groups. The decrease was largest among younger women (− 54%), and lesser among those aged 70 or over (− 22%). Of all hysterectomies, 24 per cent in 2001–2002 and 40% in 2017–2018 were performed for women aged 60 or older.

**Fig. 1:**
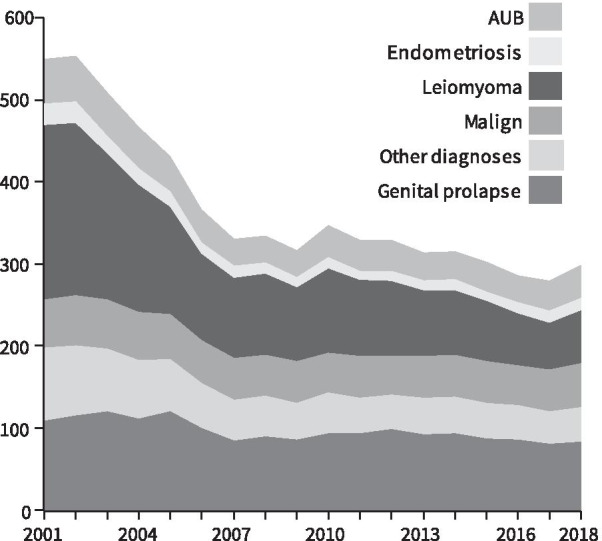
Age adjusted hysterectomy rates by indication in Finland 2001–2018. Rates calculated per 100,000 person years (*AUB* abnormal uterine bleeding)

From 2001 to 2018 age-adjusted hysterectomy rate decreased in all indications (Fig. 1). In the beginning of the study period, leiomyoma was the most common indication followed by uterine prolapse and malignancy. Hysterectomies performed due to leiomyoma decreased by 71 per cent during the study period, leaving uterine prolapse as the most common cause in 2017–2018. The rate of hysterectomies performed for leiomyoma decreased from 212/100,000 py in 2001–2002 to 61/100,000 py in 2017–2018, accounting for the largest part of decline in the total rate. Compared to other indication groups, the decrease in rates was less pronounced in hysterectomies due to uterine prolapse (change − 27%) and malignancy (change − 14%). Genital prolapses and malignancies are markedly more common in postmenopausal women than in women of fertile age, which explains that there is less reduction in hysterectomy in the age group of older women. Respectively, conservative and hysteroscopic treatment methods are increasingly being used for AUB, leiomyoma and endometriosis.

**Fig. 2:**
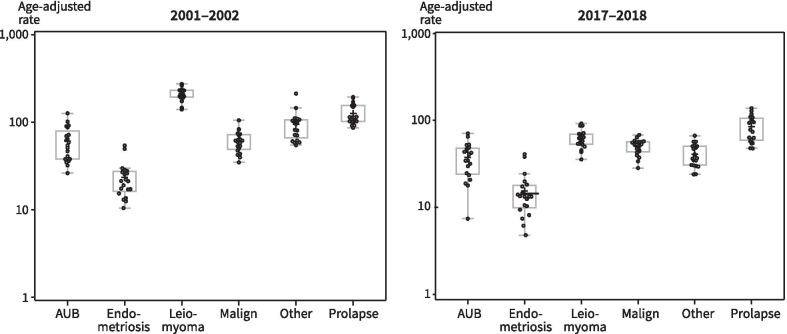
Regional variations in indications for hysterectomy in Finland, age-standardised rates per 100,000 person years across hospital districts in 2001–2002 and 2017–2018

To examine regional differences from 2001–2002 to 2017–2018 we investigated variations in hysterectomy rates in the 20 hospital districts. Differences across regions were observed in total hysterectomy rates and all hysterectomy indications as suggested by Fig. 2, in which each dot represents one hospital district. In 2001–2002 total rates of hysterectomies ranged from 405/100,000 py to 861/100,000 py, while in 2017–2018 the respective figures were 163/100,000 py, and 408/100,000 py.

During the whole study period, SCVs for indications fluctuated from year to year (data not shown). When comparing figures for indications in the beginning and the end of the study period, the SCV for AUB and endometriosis increased substantially, probably due to outliers caused by declining rates and differing age structure. For other causes, the changes were relatively small (Table 2).

**Table 2:** Systematic component of variation (SCV) between health care regions per diagnostic category in 2001–2002 and 2017–2018 and multilevel model based correlation with time between 2001 and 2018

	SCV(*100)	SCV(*100)	Correlation with time	
	2001–2002	2017–2018		
*Indication*				
Leiomyoma	0.9	2.3	− 0.93	< .0001
Malignancy	3.0	1.3	− 0.95	< .0001
Genital prolapse	1.7	1.3	− 0.71	0.0005
Endometriosis	1.2	18.5	− 0.88	< .0001
Abnormal uterine bleeding	4.7	20.3	− 0.89	< .0001
Other diagnoses	4.0	4.1	− 0.97	< .0001
All	1.5	2.1	− 0.84	< .0001

*SCV* systematic component of variation

Regional autocorrelations mainly suggest consistency in regional patterns by indication between 2001–2002 and 2017–2018 (data not shown). The autocorrelation averaged over the indications ranged from 0.18 to 0.67 between hospital districts, with an average of 0.50. This suggests that most regions that have been above (or below) the national average on some time-point have stayed high (or low) in consequent years. However, substantial differences in autocorrelations for individual indications among regions were observed. The average autocorrelation varied from 0.31 (malign causes and endometriosis) to 0.50 (uterine leiomyoma). These results indicate that consistency in medical patterns depended on indication.

Table 2 further presents results of analysis for association between regional variation and time. The estimated trend for indications was negative and statistically highly significant (*p* < 0.001) in all cases. For all hysterectomies the correlation coefficient was − 0.84 and the correlation was smallest in genital prolapse (− 0.71). These results indicate increased regional convergence in all hysterectomy indications.

## Discussion

In this study we found a change in hysterectomy rates for all indications in Finland from 2001 to 2018 along with a substantial decrease in total hysterectomy rates. During the study period regional variation in hysterectomy practices decreased as we found a statistically significant convergence of hysterectomy rates for all indications. Despite this development, variation across hospital districts still existed in 2017–2018. Historically, hysterectomy rates have been high in Finland but the decline in 2000s was rapid and more substantial when compared to findings reported from other countries [15]. The rate is suggested to currently be similar to Sweden, but higher than e.g. in Denmark [19]. However, direct comparisons with rates from other countries are not possible due to relatively large differences in study periods, selection of indications, surgical procedures and age groups that are included across studies as well as in international databases.

The overall decrease in rates could be attributed mainly to the decrease in hysterectomies performed for leiomyoma as it was the leading indication at the beginning of the study period. The decrease in proportion of operations for leiomyoma and increased proportion for prolapse since the 1990s is consistent with observations from Sweden [20] and Denmark [4]. Compared to our results, in Denmark abnormal uterine bleeding has accounted for larger part of hysterectomies and the share has even increased in the 2000s [4]. Also in the US, the proportion of operations due to leiomyoma has decreased but that of abnormal uterine bleeding increased [5]. However, dissimilarity in indications between countries may reflect differences in diagnostic practices more than those in morbidity and possible differences in coding practices may have influenced the differences between countries.

We found variation across regions both in overall hysterectomy rates and indications for these. Regional variation in indication specific rates has earlier been reported from Germany [21], Australia [22] and the Netherlands [23]. Although variation in medical practices is widely known, changes in regional variation within one country have been a focus less frequently. In a Dutch study, Hanstede et al. [23] reported decreases in temporal and regional differences in hysterectomy indications from 1995 to 2005, in Germany, Stang et al. [21] reported regional variation in 2005–2006 and the Australian Hysterectomy Clinical Reference Group reported similar findings from 2008 to 2016–2017 [22]. Westert et al. [2] investigated practice patterns in several common medical conditions and found increased regional convergence over time. These results are consistent with our findings indicating decreasing trend in regional variation.

The changes in hysterectomy practices and convergence found in hysterectomy rates are probably a result of several factors. Firstly, in 2005 the Finnish guidelines for operative treatment of abnormal uterine bleeding, leiomyoma, endometriosis and prolapse were introduced and this may have unified clinical practices [24]. Secondly, in the beginning of the 2000s results from the Finnish RCT study comparing hysterectomies with levonorgesterol releasing intrauterine devices for treating menorrhagia were published [25]. The study results also influenced the national clinical guidelines for the treatment of excess menstrual bleeding underlining pharmaceutical treatment of menorrhagia [26]. Nonetheless, it is unclear whether use of more conservative treatments enables women to avoid hysterectomy in the long term or merely delays it [27, 28]. In general, variations in medical practices may result from patient related, physician related or practice environment related factors [3]. Although differences in disease burden across the population may contribute to the variation found between regions, it is unlikely that hysterectomy related risk factors or morbidity would differ sufficiently across hospital districts to account substantially for the differences observed in this study. It has been shown that socioeconomic position may have influence on hysterectomy rate [29, 30]. However, this is unlikely to be the reason for the regional variation found.

A major strength of our study was that we used individual-level data on hospital use among all female residents of Finland over a period of 18 years derived from the Hospital Discharge Register, the quality and coverage of which has been reported to be, in general, good. A systematic review reported that more than 95% of discharges could be identified from the register and that the positive predictive value, i.e. the proportion of register-detected cases that are confirmed to be true-positives according to external data varied between 75 and 99% for common diagnoses [31]. However, we cannot exclude the possibility that indication was miscoded in a small number of cases or potential differences in coding practices. Further, since AUB and leiomyoma occur in the same patients and there is likely to be variation in which one is recorded as the first diagnosis, they could also be considered together.

The statistical models used enabled us to summarize various aspects of the data efficiently. In Finland patients are assigned to hospital districts based on their place of residence and the use of health services across regional boundaries is relatively rare. Since hysterectomies are not performed in outpatient settings, the hospital discharge data can be considered to accurately reflect the operation rates in this country. Hysterectomies for benign diseases are often associated with more than one diagnosis, and the decision to operate may depend on the combination and severity of indications presented. A limitation of our study is that, we did not take secondary diagnoses into consideration.

A potential limitation is that the analysis takes into account the age structure of the different patient populations but lacks data on other patient characteristics likely to affect clinical decision-making, e.g. patients’ reproductive history, prior surgical procedures and other health related factors. Also, age structures in operations differ by indication with younger patients in AUB and endometriosis and we were not able to examine patients aged 25–39 years and 40–49 years separately as the numbers of cases did not enable it while we acknowledge that the reasons for hysterectomy can differ before perimenopausal period. Notwithstanding, we preferred using uniform standard population for comparability reasons. Further, we did not have data on medication or intrauterine device use. Further research is needed in the development of incidence and treatment of female malignant conditions. As we did not have data on the incidence of ovarian cancer, cervical cancer, and uterine cancer we could not analyse them in more detail. Neither did we have data on patients’ preferences, which is important for operation decisions in discretionary surgery. Thus, our data did not allow us to differentiate variation occurring because of differences in patient needs i.e. warranted variation from unwarranted variation. However, it is plausible that the decrease of variation in hysterectomy rates seen in this study has focused on unwarranted variation. Based on this assumption at least part of the earlier variation in use of hysterectomy has been unjustified by patient needs.

## Conclusions

The findings of this study show major changes in hysterectomy related medical practices in Finland from 2001 to 2018 and the concurrent increase in regional convergence across health care regions. These results demonstrate that changes in medical practices and more uniform use of hysterectomy across regions are possible to achieve. Despite the positive trend, regional variation in rates, indications and surgical approaches still exists, which may suggest inequity in availability of healthcare and non-optimal use of health care resources. More in-depth studies are needed by indication taking into account the seriousness of the disease, availability of other treatment options, patient preferences as well as comorbidity.

## Data Availability

The data that support the findings of this study are available from the Finnish Institute for Health and Welfare and Statistics Finland but restrictions apply to the availability of the data used under license for the current study, and so they are not publicly available due to Finnish data protection legislation. According to the legislation, register authorities give permissions to use register data including sensitive individual information (e.g., health data) to study specified research questions to named individuals who have signed a pledge of secrecy and they are not permitted to forward it to other researchers. Other researchers can apply for the data from the Health and Social Data Permit Authority Findata. Findata handles the data permit applications concerning Finnish Institute for Health and Welfare registers (the Care Register for Health Care for operations and diagnoses in the current study), and Statistics Finland data when combined to other registers (population at risk and demographics) see https://www.findata.fi/en/services/data-requests/.

## Abbreviations

AUB: Abnormal uterine bleeding
py: Person years
SCV: Systematic component of variation
